# Satellite-derived mineral mapping and monitoring of weathering, deposition and erosion

**DOI:** 10.1038/srep23702

**Published:** 2016-03-30

**Authors:** Thomas Cudahy, Mike Caccetta, Matilda Thomas, Robert Hewson, Michael Abrams, Masatane Kato, Osamu Kashimura, Yoshiki Ninomiya, Yasushi Yamaguchi, Simon Collings, Carsten Laukamp, Cindy Ong, Ian Lau, Andrew Rodger, Joanne Chia, Peter Warren, Robert Woodcock, Ryan Fraser, Terry Rankine, Josh Vote, Patrice de Caritat, Pauline English, Dave Meyer, Chris Doescher, Bihong Fu, Pilong Shi, Ross Mitchell

**Affiliations:** 1 CSIRO Mineral Resources, Australian Resources Research Centre, 26 Dick Perry Avenue, Kensington, Western Australia, 6151, Australia; 2 Geoscience Australia, GPO Box 378, Canberra, ACT, 2601, Australia; 3 School of Mathematical and Geospatial Sciences, GPO Box 2476, Melbourne, Vic 3001, Australia; 4 Jet Propulsion Laboratory/California Institute of Technology, Pasadena, CA, USA; 5 Japan Space Systems, 3-5-8 Shibakoen, Minatoku, Tokyo, 105-0011, Japan; 6 Geological Survey of Japan, National Institute of Advanced Industrial Science and Technology, Central 7, 1-1-1, Higashi, Tsukuba, 3058567, Japan; 7 Nagoya University, Department of Earth and Environmental Sciences, Graduate School of Environmental Studies, D2-1 (510) Furo-cho, Chikusa-ku, Nagoya, 464-8601, Japan; 8 CSIRO Data61, Leeuwin Centre, Brockway Road, Floreat Park, Western Australia, 6014, Australia; 9 CSIRO Mineral Resources, Life Sciences Centre, Riverside Corporate Park, 11 Julius Avenue, North Ryde, NSW, 2113, Australia; 10 CSIRO Mineral Resources, Computer Science & IT Building, 108 North Road, Canberra, ACT, 2601, Australia; 11 Research School of Earth Sciences, The Australian National University, Canberra, ACT 2601, Australia; 12 USGS/EROS, 47914, 252^nd^ Ave. Sioux Falls, South Dakota, 57198-0001, USA; 13 Chinese Academy of Sciences, Institute of Remote Sensing and Digital Earth, 9 Dengzhuang South Road, Haidian District, Beijing 100094, P.R. China; 14 CSIRO Oceans and Atmosphere, Forestry House, Banks Road, Yarralumla, ACT, 2601, Australia

## Abstract

The Earth’s surface comprises minerals diagnostic of weathering, deposition and erosion. The first continental-scale mineral maps generated from an imaging satellite with spectral bands designed to measure clays, quartz and other minerals were released in 2012 for Australia. Here we show how these satellite mineral maps improve our understanding of weathering, erosional and depositional processes in the context of changing weather, climate and tectonics. The clay composition map shows how kaolinite has developed over tectonically stable continental crust in response to deep weathering during northwardly migrating tropical conditions from 45 to 10 Ma. The same clay composition map, in combination with one sensitive to water content, enables the discrimination of illite from montmorillonite clays that typically develop in large depositional environments over thin (sinking) continental crust such as the Lake Eyre Basin. Cutting across these clay patterns are sandy deserts that developed <10 Ma and are well mapped using another satellite product sensitive to the particle size of silicate minerals. This product can also be used to measure temporal gains/losses of surface clay caused by periodic wind erosion (dust) and rainfall inundation (flood) events. The accuracy and information content of these satellite mineral maps are validated using published data.

The Earth’s regolith comprises all surface materials between “fresh rock and fresh air” where oxidation, hydration, biological and mechanical processes have caused some degree of mineralogical change (weathering) and porosity generation of underlying rock^1^,^2^. Parent rock, tectonics, climate, landform, groundwater, vegetative groundcover and time all contribute to the composition and architecture of the regolith^3^. Progressive weathering is generally associated with increased development of volatiles yielding minerals like hydroxyl-bearing clays (for example, illite, montmorillonite and kaolinite) as well as decreased levels of mobile cations (e.g. Ca, Mg, K and Na) as a consequence of hydrological leaching^3^,^4^,^5^,^6^.

The intensity of weathering has been gauged using laboratory geochemical techniques like loss-on-ignition (LOI)^7^ and the chemical index of alteration^5^ {CIA = [Al_2_O_3_/(Al_2_O_3_ + CaO* + Na_2_O + K_2_O)] × 100; where CaO* is the amount of CaO incorporated in silicate minerals}. The CIA was designed to estimate the amount and composition of clay minerals based on the assumption that kaolin [Al_2_Si_2_O_5_(OH)_4_] is associated with intense weathering that is typical of warmer, wetter conditions, such as the subtropical monsoonal belt. In contrast, illite [(K,H_3_O)(Al,Mg,Fe)_2_(Si,Al)_4_O_10_[(OH)_2_·(H_2_O)] and montmorillonite [(Na,Ca)_0.33_(Al,Mg)_2_(Si_4_O_10_)(OH)_2_·*n*H_2_O] are indicative of less intense weathering typical of cooler, temperate conditions^5^,^6^,^8^. Not included in the CIA is silicon (Si) which is abundant in the Australian regolith^4^ as quartz (SiO_2_). Quartz grains are generally equant in shape and large in diameter (>20 μm but <2000 μm; called “sand size”) and so are less vulnerable to erosion by wind^9^ compared with clay minerals, which typically have a finer grain size (<2 μm; called “clay size”) and platy habit.

Australia is a flat, dry, deeply weathered continent reflecting its low rates of erosion and long-lived tectonic stability associated with its mosaic of Precambrian blocks^1^. Approximately 80% of Australia is covered by a thick blanket of regolith that largely comprises clays, iron oxides and quartz^1^,^4^. This deep weathering began from at least 45 Ma where tropical conditions extended down to a latitude 30° south. From 10 Ma this monsoonal belt began to move northward exposing Australia’s inland regions to increasingly arid conditions and resulting in the formation of extensive deserts^10^. Sandy dune-fields like the Simpson Desert result from the preferential loss of surface clay materials by wind erosion. Today, Australia’s drylands (rainfall/evaporation < 0.65) generate the largest source of dust in the southern hemisphere contributing 5–15% of the global dust emissions^11^. This dust is potentially impacting on human health^12^, the sustainability of agricultural soils^13^,^14^,^15^, episodic oceanic algal blooming^16^ and atmospheric aerosol scattering and climate change^17^,^18^.

Airborne geophysical methods like gamma-ray spectrometry^19^ are currently used in Australia to measure clay information^20^ and map the regolith^21^. Reflectance/emission spectroscopy using field, airborne and spaceborne systems can also provide information about clays, quartz and particle size^22^,^23^,^24^,^25^,^26^,^27^. There have been more than two hundred Earth observation (EO) reflectance/emission satellite missions though only one of these EO sensors to date was designed specifically and operated successfully for global mapping of mineral information, namely ASTER (Advanced Spaceborne Thermal Emission and Reflection Radiometer)^28^. This Japanese-US system was launched in December 1999 as part of a multi-sensor payload onboard the USA’s Terra space platform for servicing NASA’s Mission to Planet Earth, now called Earth Science Enterprise^29^.

ASTER is a polar orbiting (<83° latitude) system with a 60 km imaging swath designed to sense across fourteen spectral bands at a pixel resolution between 15 and 90 m. These spectral bands include: (a) three in the visible and near infrared (VNIR, 0.4 to 1 μm) wavelength region where green vegetation and iron oxides have diagnostic spectral features; (b) six in the shortwave infrared (SWIR, 1–2.5 μm), where clay minerals have diagnostic features related to the vibration of hydroxyl groups; and (c) five in the thermal infrared (TIR, 8–12 μm), where non-hydroxyl bearing silicate minerals like quartz have diagnostic features though particle size also has a pronounced effect^24^. The ASTER global land surface mapping program is into its sixth iteration, though the SWIR module failed permanently in 2008. The first public, continent-wide ASTER mineral mosaics were generated for Australia in 2012^30^,^31^ using 3500 satellite images collected between 2000 and 2007.

This paper focuses on three of the available seventeen published ASTER geoscience maps^30^ plus introduces a new one designed for measuring water content and/or particle size (see Methods). These four ASTER SWIR and TIR mineral products focus on clay, water, quartz and particle size information and are validated using published field sample data as well as meteorological, geophysical and other satellite data. Their value for mapping and monitoring the effects of weathering, erosion and deposition in the context of variable vegetation cover, climate and tectonic processes is assessed before a final perspective on the future of mineral mapping and monitoring of the Earth using integrated (VNIR-) SWIR-TIR imaging systems is outlined.

## Results

**Figure 1:**
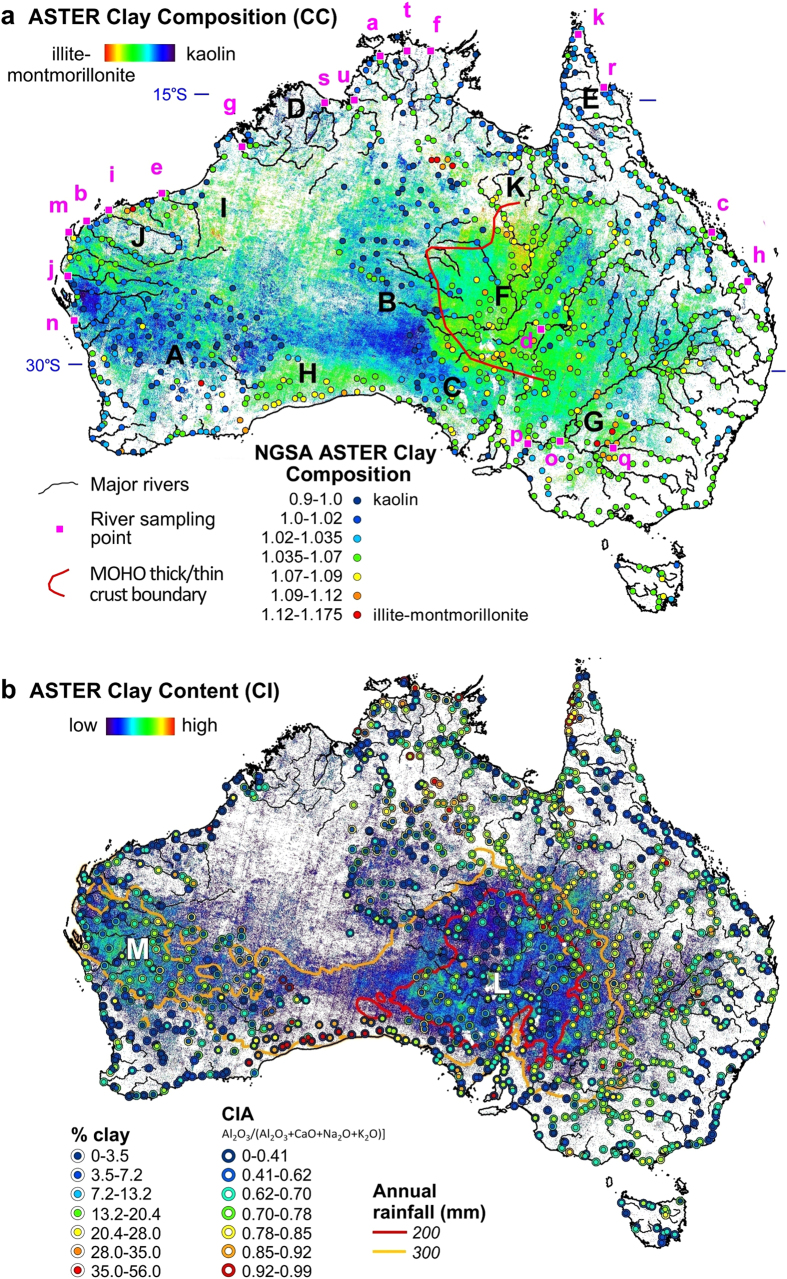
ASTER clay maps with associated validation data. (**a**) ASTER Clay Composition (CC) map (background) overlain by: (i) NGSA field sample reflectance data convolved and processed to ASTER CC response; (ii) river sampling points^35^; (iii) approximate boundary of thin crust^52^; and (iv) selected localities, namely: A – Yilgarn Craton, B – Musgrave Block, C – Gawler Block, D – Kimberley Block, E – Cape York Penisula, F – Lake Eyre Basin, G – Murray-Darling Basin, H – Eucla Basin, I – Canning Basin, J – Pilbara Block, K – Mount Isa Block. (**b**) ASTER Clay Content (CI) map (background) overlain by: (i) NGSA field sample data for % clay and CIA [Al/(Al + Ca + Mg + K + Na)]^33^,^34^; (ii) isolines of rainfall averages for 1996–2005^47^; and (iii) selected localities, namely: L – Lake Eyre Basin and M – Gascoyne. Figure created using the following software: ENVI (4.3, http://www.exelisvis.com/ProductsServices/ENVIProducts.aspx), ARCMAP (10.2.2, http://www.esri.com/software/arcgis/arcgis-for-desktop), EXCEL 2013 (http://www.microsoftstore.com/store) and COREL Photopaint (7, http://www.corel.com/au).

**Figure 2:**
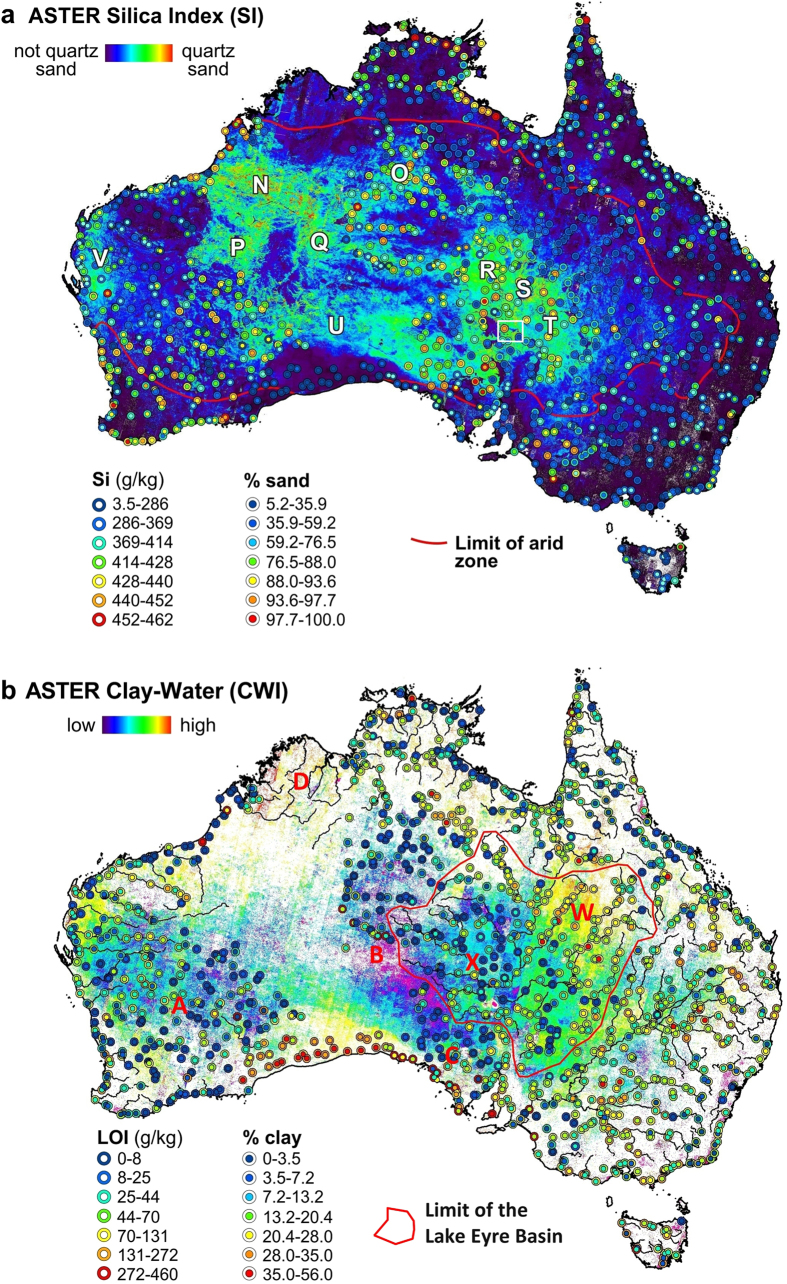
ASTER quartz sand and clay-water maps with associated validation data. (**a**) ASTER Silica Index map overlain by: (i) field sample NGSA data for Si (geochemistry) and % sand (particle size)^33^,^34^; (ii) limit of arid zone^1^; and (iii) sandy deserts, namely: N – Great Sandy, O – Tanami, P – Little Sandy, Q – Gibson, R – Simpson, S – Sturt Stony, T – Strzelecki, U – Great Victoria and V – northwest dune-fields; and (iv) detailed study area of Fig. 3a (white rectangle). (**b**) ASTER Clay-Water Index (CWI) map (background) overlain by: (i) field sample NGSA data for LOI and % clay^33^,^34^; (ii) extent of the Lake Eyre drainage basin (red polygon); and (iii) selected localities referred to in the text. Figure created using the following software: ENVI (4.3, http://www.exelisvis.com/ProductsServices/ENVIProducts.aspx), ARCMAP (10.2.2, http://www.esri.com/software/arcgis/arcgis-for-desktop), EXCEL 2013 (http://www.microsoftstore.com) and COREL Photopaint (7, http://www.corel.com/au).

The four ASTER mineral maps selected for mapping Australia’s regolith include: (i) “Clay Composition” or “CC” (also called AlOH Group Composition^30^), which was designed to measure the geometry of the dioctahedral clay absorption at 2.2 μm and depicts kaolinite as cooler tones and illite-montmorillonite as warmer tones (Fig. 1a); (ii) “Clay Content” or “CI” (also called AlOH Group Content^30^), which is a measure of the hull-continuum-depth of the clay absorption at 2.2 μm with areas of detectable clay showing colour (Fig. 1b); (iii) “Silica Index” or “SI”, which is sensitive to both the intensity and wavelength position of the silicate reststrahlen feature and is driven by the bulk SiO_2_ content^23^ and particle size^24^ with areas of high quartz sand content mapped as warmer tones and clay-rich as cooler tones (Fig. 2a); and (iv) “Clay-Water Index” or “CWI” (see Methods), which measures the slope of the short-wavelength wing to the fundamental water stretching vibration absorption at 2.7 μm such that water-rich areas yield warmer tones (Fig. 2b). The accuracy of these ASTER mineral products is assessed using the National Geochemical Survey of Australia (NGSA)^32^,^33^,^34^ and published studies of the mineralogy of suspended river loads during flood events^35^ and outboard marine sediments^36^,^37^.

### ASTER validation using the NGSA

The NGSA is a public suite of physicochemical data (LOI, major/minor/trace element geochemistry and particle size) measured from unconsolidated, flood-overbank samples collected at 0–10 cm and ~60–80 cm depths from over 1000 river catchments across Australia^32^,^33^,^34^. High resolution laboratory VNIR-SWIR-TIR reflectance spectra of these NGSA samples were measured before convolution to simulate the ASTER responses (see Methods). Statistical assessment of a random NGSA sample subset (N = 165) focused on relationships between the ASTER geoscience products (CC, CI, CWI and SI) and comparable physicochemical parameters, chiefly LOI, % clay and % sand. The results (Table 1) show that: (i) ASTER CC is significantly correlated (@90% confidence interval) with LOI, % clay and inversely with % sand; (ii) ASTER CWI is correlated with % clay and to a lesser degree LOI and inversely correlated with % sand; (iii) ASTER SI is correlated with % sand as well as Si (R^2^ = 0.64, N = 165) and inversely correlated with % clay and LOI; and (iv) no significant correlations with respect to LOI, % clay and/or % sand for the ASTER CI, CIA and the gamma-ray spectrometry analogues K, Th and U.

The ASTER CC image is plotted with the NGSA ASTER CC in Fig. 1a which shows high spatial similarity across the continent. Areas mapped as rich in kaolin (cooler tones) include the Yilgarn (“A”), Musgrave (“B”), Gawler (“C”) and Kimberley (“D”) Precambrian cratons as well as Cape York Peninsula (“E”) while illite-montmorillonite (warmer tones) is developed over Lake Eyre (“F”), Murray-Darling (“G”), Eucla (“H”) and Canning (“I”) basins as well as the Pilbara (“J”) and Mount Isa (“K”) Precambrian blocks. Note that these regions span arid to sub-tropical climatic regimes as well as different groundcover conditions ranging from dense green vegetation, including forests, to areas of sparse dry plant materials, namely grasses and shrubs, to completely exposed regolith surfaces.

The significant correlation between the NGSA ASTER CC and LOI (Table 1) is consistent with a published relationship between water content and % clay in soils^38^, which is also apparent in the NGSA data through the association between % clay and LOI (R^2^ = 0.29, N = 1069). Clay mineralogy is expected to be a key driver in understanding a given sample’s water content as there is a 100-fold increase in absorption/adsorption of water as the clay mineralogy changes from kaolinite to illite to montmorillonite, which relates to their respective cation-exchange capacities and typical crystallite sizes^39^. The ASTER CC is also correlated with Si (R^2^ = 0.35, N = 165) and to a lesser degree Si/(Si + Al) (R^2^ = 0.28, N = 165), which suggests a role for silicic acid in the formation and stability of kaolin^40^. Note that the geochemical index Al/(Si + Al) is used by other workers^41^ as an indicator for estimating the clay composition of dust and for tracking its possible source.

Similar to the laboratory results (Table 1) and other studies^27^ there is little correlation between the ASTER CI and NGSA % clay (Fig. 1b), which can be explained by: (i) clay size content is not the same as clay mineral content; (ii) different clay minerals have different 2.2 μm absorption depths; (iii) the lack of an ASTER band at ~2.1 μm to adequately constrain the short-wavelength hull-continuum of the clay 2.2 μm absorption, with kaolinite being the most adversely affected because of a doublet absorption at 2.16 μm; (iv) complex scattering behaviour as silicate particle sizes decreases <75 μm^22^; (v) mixing with optically “transparent” grains, especially quartz, or relatively opaque phases, like carbon black^22^, which affects the path-length of the transmitted/reflected light^42^; and (vi) the effects of green and/or dry vegetation cover^25^.

The ASTER CI shows a close relationship with the rainfall pattern (Fig. 1b) with the arid zone (<300 mm annual rainfall) showing the greatest intensity of 2.2 μm absorbing clay minerals. This association is presumably related to a low amount of vegetative groundcover (see Methods), with areas of greatest exposure including the Lake Eyre Basin (“L”) and the Gascoyne region of Western Australia (“M”). These areas are also major sources of dust activity^43^,^44^. That is, the ASTER CI is potentially useful for mapping clay-rich regolith with little or no groundcover and thus is at high risk to wind erosion (dust activity).

The correlation between the NGSA ASTER SI with % sand (and inverse correlation with % clay - Table 1) as well as with Si underlines the importance of quartz in the development of Australia’s regolith and the potential of using the TIR to measure silicate mineral particle size^24^. These laboratory-based correlations are also mirrored in the satellite ASTER SI map of Australia (Fig. 2a), which shows close spatial association with both the NGSA % sand and Si 0–10 cm sample data within the arid zone (red polygon). Warmer tones in the ASTER SI map accurately delineate all the quartz-sand deserts across Australia (N to V in Fig. 2a)^10^. Beyond this arid zone the correlation diminishes presumably because of the effect of vegetation cover which was not compensated for in the Version 1 ASTER maps^30^ (see Methods).

The new ASTER CWI map (Fig. 2b) shows spatial similarity to the embedded NGSA LOI and % clay field sample data especially for drier parts of the continent such as the region between the Yilgarn in Western Australia (“A”) to the channel country of Queensland (“W”). However, there is departure for higher rainfall (e.g. seaboard) and/or humid (e.g. subtropical north) areas. This departure can be explained by the ASTER CWI also being sensitive to atmospheric water vapour which was not adequately corrected for in the ASTER Version 1 pre-processing^30^,^31^ (see Methods).

For drier areas, a combination of the ASTER CC and CWI maps (Figs 1a and 2b) enables the discrimination of the clay minerals, illite from montmorillonite, assuming that montmorillonite is associated with relatively higher amounts of absorbed/adsorbed water^39^. Using this assumption, the clays across the Lake Eyre basin (red polygon in Fig. 2b) can be divided into water-rich montmorillonite in the eastern half (“W”), which is consistent with extensive cracking soils in the braided channel country^45^, from illite in the western part of the basin (“X”), which is consistent with the high K radiometric response^19^ of alluvial sediments shedding off the Musgrave (“B”) and Gawler (“C”) Blocks. Note also the water-poor, kaolin-rich area between the Musgrave and Gawler Blocks (“B” and “C” in Figs 1a and 2b) and how it contrasts with the water-rich, kaolin-rich Kimberley Block (“D” in Figs 1a and 2b), which is consistent with an uncorrected water vapour effect in the current ASTER CWI product.

The NGSA results (Table 1) show that % clay and LOI are not correlated with the gamma-ray geochemical analogues K, Th or U. Thus even though local-scale correlations may exist^20^, use of gamma-ray spectrometry for universal mapping of clay information is problematic. It also explains why an earlier predicted map of Australia’s clay mineral composition^46^, which relied on statistical modelling primarily of airborne gamma radiometric data^19^, is different to that measured using the ASTER CC and CWI (Figs 1a and 2b), especially for kaolin-rich areas.

### ASTER validation using fluvial-marine sediment data

The clay mineralogy measured using laboratory X-Ray diffraction (XRD) of suspended river sediment samples^35^ (locations labelled “a” to “u” in Fig. 1a) is compared with the ASTER CC responses calculated using the means of the corresponding catchment areas. The results (Table 2) show correlation between the catchment CC and the river point-sample, % kaolinite and % illite + % montmorillonite. This positive result suggests that for a given catchment, the ASTER CC can be used to gauge the clay composition of the sediments carried by the associated river network. These river sediments are deposited in fringing (10 to 200 km) continental shelf marine environments where published studies^6^,^36^,^37^ show clay mineralogy consistent with the ASTER CC map. For example sediments off the Pilbara (“J” in Fig. 1a) coast are characterised by illite which is consistent with both the illite signature of the suspended sediments in the Ashburton, De Grey, Fortescue and Gascoyne Rivers and the ASTER CC map (j, b, e, i, j and m in Table 2 and Fig. 1a). In contrast, kaolin-rich sediments offshore from the Murchison River (n in Fig. 1a) are consistent with kaolinite-rich sediments of the Murchison River (Table 2) and its catchment surface mapped using the ASTER CC. More distal marine sediments off the Australian continental shelf (up to 3000 km) show a pattern of kaolinite in the Indian Ocean whereas illite is relatively common in the southwestern Pacific Ocean^37^. This pattern mirrors the ASTER CC (Fig. 1a) though the greater distances indicate that dust transport is likely to be responsible for generating these marine clay sediments which is consistent with wind patterns^43^,^44^.

### Monitoring surface clay loss with ASTER TIR

**Figure 3:**
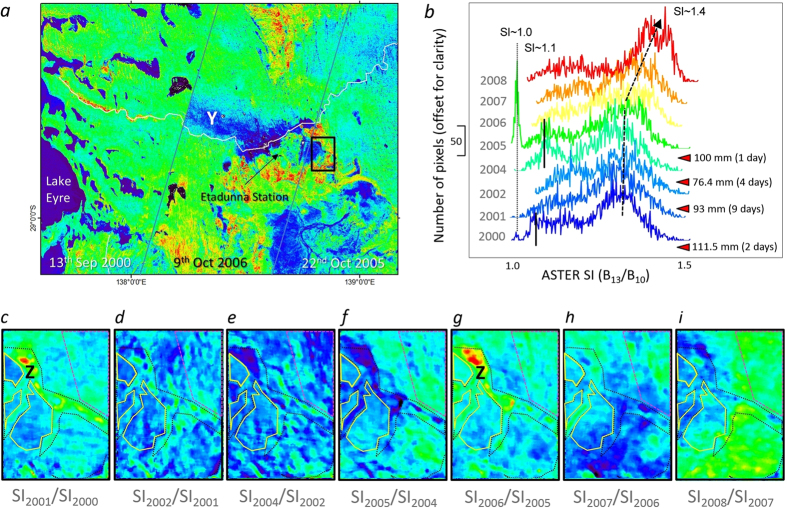
Temporal variations in ASTER SI over Cooper Creek, Lake Eyre basin. (**a**) Detail of the published ASTER SI mosaic^30^ (Fig. 2a; white rectangle) for an area of Cooper Creek (white line) which drains into Lake Eyre. Colour scheme is the same as Fig. 2a. Image is overlain by: (i) ASTER satellite track boundaries (grey lines) of three dates of ASTER images (dates shown); (ii) evidence for a dust trail “Y”; (iii) Etadunna Station, which is the site for ground meteorological observations; (iv) detailed area (black rectangle) for temporal study of ASTER SI. (**b**) Stacked histograms of ASTER SI data (offset and coloured for clarity) for eight dates of imagery from 2000 to 2008 for the detailed study area. Also shown are: (i) periods of intense rainfall; and (ii) SI positions for clay and sand size material, which shows an apparent increase in sand size material from 2006 to 2008. (**c**–**i**) changes in SI response for consecutive dates of ASTER imagery from 2000 to 2008 for the detailed study area (~9 by 13 km) calculated as a ratio of the following image date. Warmer response equals a relative loss of clay or gain of sand whereas a cooler response represents a relative gain of clay or loss of sand compared to the previous year/image. Drainage lows, including ephemeral lakes, are shown by black dotted lines. Aeolian sand dunes are show by a magenta dashed lines and rocky surfaces are shown by a yellow solid lines. Figure created using the following software: ENVI (4.3, http://www.exelisvis.com/ProductsServices/ENVIProducts.aspx), ARCMAP (10.2.2, http://www.esri.com/software/arcgis/arcgis-for-desktop), EXCEL (2013, http://www.microsoftstore.com) and COREL Photopaint (V7, http://www.corel.com/au).

The highest correlation with NGSA particle size is provided by the TIR-based ASTER SI (Table 1). However, is there sufficient sensitivity with these ASTER TIR bands to measure clay loss or gain caused by short (~hours to days) to long (years) term erosional/depositional processes? To test this possibility, an area along Cooper Creek east of Lake Eyre (Fig. 3a) was selected because it: (i) showed mismatches in the ASTER Version 1 SI mosaic (Y in Fig. 3a) related to the different dates of satellite overpasses; (ii) comprises annual, cloud-free ASTER images for the period 2000 to 2008; (iii) spans a diversity of regolith surface types, including ephemeral drainage, aeolian sand dunes and rocky ground; (iv) availability of meteorological data^11^,^47^; and (v) spans a history of variable dust and rainfall activity^11^,^47^,^48^.

The three satellite overpasses (dated 13^th^ September 2000, 22^nd^ October 2005 and 9^th^ October 2006) used in the published ASTER SI mosaic^30^ and spanning the ephemeral Cooper Creek (white line Fig. 3a) appear to be seamless except for a 110–290° trending, 20 km wide band only present in the 9^th^ October 2006 satellite path (Y). This clay-rich band (interpreted by the cool tones in the ASTER SI), which is not evident in either earlier or later dates of ASTER imagery for the same path/area, cross-cuts sand dunes to the west-northwest before truncating against the 13^th^ September 2000 scene. This clay band also shows a decreasing SI gradient (increasing clay content) towards Cooper Creek where it terminates (originates).

Meteorological data recorded from the weather station at Etadunna Station^47^on the morning of the 9^th^ of October 2006 shows an average (over 9 hours) wind speed of 33 km/hr from the direction 134°. This wind event is towards the upper limit of wind activity for this region between 2000 to 2008 and thus could be responsible for this trail of clay material in the 9^th^ October 2006 ASTER SI image (Fig. 3a). However, the coincident timing with the satellite overpass means that a component of the detected dust trail may also have been airborne provided the entrained dust particle size was sufficiently large (~10 μm diameter). An even stronger and more closely aligned wind event occurred on the 13^th^ July 2006, which had an average (21 hours) speed of 40.7 km from a direction of 108°. Note that other satellite observations^48^ recorded numerous dust activity events originating from this section of Cooper Creek during 2005/2006.

Ground-based nephelometer aerosol data were recorded 100 km to the east-southeast at Tinga Tingana^11^. These data show that dust activity was higher in the summer months from 2003/2004 to 2007/2008 and that 2005/2006 was the most intense. Similarly, total rainfall at Etadunna Station^47^ in the 12 months preceding the 9^th^ October 2006 was only 76.4 mm, which was significantly less than the 2000–2008 annual average of 129.5 mm. These meteorological data support the hypothesis that the cross-cutting clay band in the 9^th^ October 2006 ASTER SI track is the result of a dust event that occurred either during the satellite overpass or more likely three months previously.

The non-linear relationship between ASTER SI and particle size (Table 1) means that the highest sensitivity for measuring any change is provided by areas richer in quartz sand content whereas clay-rich surfaces provide the least sensitivity. The question is whether ASTER has sufficient resolution to measure short-term changes in clay content over clay-rich surfaces, such as ephemeral drainage lows which are a known major source of dust^48^. To help address this question, a sub-area (10*14 km; black rectangle in Fig. 3a) was selected for temporal analysis because it provided the most temporally complete (2000–2008) and cloud-free set of ASTER imagery and comprises key regolith types, namely: (a) ephemeral lake and drainage lows (black dotted lines in Fig. 3c–i); (b) rocky pavements (yellow lines in Fig. 3c–i); and (iii) aeolian sand dunes (magenta dashed lines in Fig. 3c–i). There were no flood events recorded^47^,^49^ for this area during the 2000–2008 period though there were four periods of significant rainfall (~100 mm) over short periods (<10 days), namely: February 2000; June 2001; February 2003; and October 2004 (Fig. 3b). These local rainfall events had the potential to cause local inundation of drainage lows and deposit clay material.

Histograms of the eight dates of subarea SI imagery (Fig. 3b) show a suite of peaks, including: (i) a sharp peak centred at SI ~ 1 for 2005 and to a much lesser degree for 2000; (ii) a broader peak centred at SI ~ 1.1 evident for most dates, especially 2000, 2004 and 2005; and (iii) a major broad peak near SI ~ 1.4 (~sand) for all dates, which appears to increase in both position (SI value ~ particle size) and relative intensity (amount of sand material) from 2005 to 2008. The relative sharpness and low value of the SI ~ 1.0 peak (Fig. 3b) is interpreted to be related to well-sorted, clay material. The appearance of this SI peak for 2005 and to a much lesser degree for 2000 follows the two most intense rainfall events, namely the 29^th^ October 2004 (100 mm over one day) and 11^th^ and 12^th^ February 2000 (111.6 mm in two days). The SI ~ 1.1 peak is interpreted to be related to more poorly-sorted, medium grained (~silt size) material. This peak also appears to follow the three most intense rainfall events (Fig. 3b). The broad SI ~ 1.4 peak is interpreted to be related to poorly-sorted, sand size material that increased in size fraction and relative abundance from 2005 to 2008.

To understand what regolith units (especially ephemeral lakes, sand dunes and rocky ground) are driving these changes in the SI histograms (Fig. 3b), ratios of temporally-consecutive ASTER SI images were calculated for the ~120 km^2^ subarea (Fig. 3c–i). These show coarsening particle size (relative loss of a finer fraction) as warmer colours. The two largest coarsening episodes occur over an ephemeral lake (“Z”) for the SI_2001_/SI_2000_ (Fig) and SI_2006_/SI_2005_ (Fig. 3g) products. Conversely, this same lake is responsible for the largest occurrences of decreasing particle size as evident in the SI_2005_/SI_2004_ (Fig. 3f) and SI_2004_/SI_2002_ (Fig. 3e) products, which also coincide with heavy rainfall events in February 2003 and October 2004 (Fig. 3b). Note that the largest gains/losses occur towards the centre of the lake. This combined with its associated well-sorted, very-fine particle size (Fig. 3b), can be explained by the large area of this lake (700 by > 1700 m). That is, silicate particles of varying size was eroded from the neighbouring higher ground during heavy rainfall events which was transported as surface runoff down into the low energy lake environment where first the coarser fraction followed by then increasingly finer fractions were deposited away from the lake margin. This energy gradient coupled with the eventual drying of the lake explains why the greatest change in the SI ratio products is towards the lake’s centre. Other workers^48^ have noted that ephemeral lakes and other drainage lows susceptible to flooding are responsible for over 50% of the dust activity in this region even though they represent <15% of the regolith surface cover. Importantly, these results show that ASTER has sufficient spatial, spectral and radiometric resolution to detect and measure these short-term gains/losses in surface particle size/distribution for a complexity of regolith unit types across local catchments (~120 km^2^).

Previous studies^48^ found that aeolian sand dunes, which account for ~33% of the exposed surface regolith, generate ~37% of the dust activity. That is, they are the second major source of dust generation after ephemeral lakes and other drainage lows susceptible to flooding. Note that these sand dunes are not pure quartz sand but a mixture of particles from finer to coarser particle size, which explains their mid-levels (green tones) in the SI image (Fig. 3a). The ASTER SI trend of coarsening sand size material from 2004 to 2008 (Fig. 3b), which corresponds to increased dust activity in the same period^11^ and low rainfall conditions across the region^47^, is shown by the SI temporal ratios to be associated with aeolian sand dunes (magenta dashed lines, Fig. 3f–i).

The most stable SI temporal ratio values (Fig. 3c–i) are associated with regolith comprising a protective surface of rock material (yellow polygons in Fig. 3c–i). This is also consistent with previous studies^48^ which found <5% of dust activity originated from rocky areas even though they comprise ~40% of the regolith surface.

## Discussion

The surface mineralogy measured using ASTER is consistent with a changing Cenozoic climate. Given kaolinite formation is an indicator for sub-tropical conditions^5^, then the northward progression of the monsoonal belt followed by low rates of erosion during the Cenozoic can explain the extensive development of kaolinite across much of Australia (Fig. 1a). In particular, the northern zone around 15 °S, which includes the Kimberley Block and Cape York Peninsula (D and E in Fig. 1a), experiences sub-tropical conditions today causing deep weathering and the generation of kaolinite and lateritic bauxite deposits^50^. The southern extent of kaolin spans the Yilgarn, Musgrave and Gawler Cratons (A, B and C in Fig. 1a) and marks the monsoonal belt from 45 to 10 Ma where it was located as far south as ~30 °S. The preservation <10 Ma of this lateritic kaolinite (and bauxite) in the western half of Australia^4^ is related to its low relief, low rainfall and hence low erosion rates^1^,^4^. Conversely, the higher relief and rainfall of the Pilbara and Mount Isa blocks (J and K in Fig. 1a), as well as the eastern Australia highlands^51^, has resulted in extensive erosion of the original lateritic regolith blanket leaving fresh rock minerals exposed at the surface, including muscovite which is spectrally similar to illite.

Recent geophysical data and derived models reveal considerable variability in the thickness of Australia’s crust down to at least the Mohorovičić discontinuity (MOHO)^52^. For example, relatively thick (>40 km) crust underlies the region spanning the Gawler, Musgrave and Mount Isa Blocks (B, C and K in Fig. 1a). This zone of Precambrian basement is juxtaposed against thin crust (~25 km) centred under Lake Eyre (F in Fig. 1a) which has been the locus for a series of overlaying Phanerozoic basins. The arcuate boundary between these regions of contrasting crustal thickness (red line in Fig. 1 s) approximates the original position of the South China Block which rifted from the Musgrave and Gawler Blocks during the break-up of Rodinia at ~800 Ma^53^. The resultant cavity has since been a depositional environment beginning with the Cambrian Warburton Basin and extending to the present-day Lake Eyre Basin (Fig. 2b).

The pronounced gradient in crustal thickness between the Precambrian blocks and the Lake Eyre basin is also a locus for earthquake activity^54^, which is presumably driven by isostatic readjustments. That is, erosional denudation of the thicker cratons causes localised, upward rebounding of the crust whereas the Lake Eyre basin overlying thin crust continues to be filled by sediments and thus sinks in the process. Such contrasting, localised isostatic adjustments also account for the inverse relationship between crustal thickness and land surface topography. That is, Lake Eyre is currently 15 m below sea level which contrasts with the topographic highs of the Musgrave (~1000 m above sea level), Gawler (~450 m above sea level) and Mount Isa (~350 m above sea level) blocks (B, C and K respectively in Fig. 1a). Isostasy-driven seismicity also explains the existence of mantle chemical signatures in the basin groundwater^55^ as well as its high heat flux^56^ via the upward migration of mantle derived fluids along seismically-active, crustal-penetrating fractures and faults.

Importantly, ASTER shows that illite-montmorillonite characterises the sediments across the Lake Eyre basin as well as other large depositional basins developed over relatively thin crust like the Eucla and Canning Basins (H and I in Fig. 1a). The presence of illite and montmorillonite reflects the importance of sedimentary provenance (for example from muscovite-rich fresh rock from the Musgrave Block) and/or the chemistry and movement of shallow groundwater.

Arid conditions post 10 Ma presumably led to a loss of ground cover and an increase in wind erosion of surface clays causing the formation of extensive sandy deserts^1^,^10^. The extent of this surface sand is easily mapped (and monitored) using the ASTER SI (Fig. 2a). However, the ASTER CC, CI and CWI (Figs 1a,b and 2b) show that these sandy deserts have very different patterns in clay mineralogy, reflecting different processes of formation. For example, the sand-rich areas over the Gawler Craton area span both kaolin- and illite-rich areas (C in Figs 1a and 2a,b). This independency underlines: (i) the unique and complementary nature of the SWIR versus TIR wavelength regions and their derived mineral products; (ii) how this diverse mineral information is diagnostic of different regolith processes; and (iii) the error in assuming that the Earth’s exposed land surface can be accounted by a single, uniform component or spectral band.

Fingerprinting the source of dust events has been a challenge to date^17^,^18^. For example, sediment deposited during a major dust storm over eastern Australia in September 2009 comprised a mixture of clay minerals, especially montmorillonite^57^. The dust’s fine particle size (>50%<10 μm) and high clay mineral content (Al/Si > 0.3)^41^ was used to infer a source from desert areas approximately 1000 km to the west of Sydney^57^. Given the importance of montmorillonite in this dust, the ASTER clay mineral maps (Figs 1a,b and 2b) indicate suitable sources between “G” and “W”. If illite or kaolinite were instead the dominant clay mineral, then likely sources are located further to the west.

A 2012 report^58^ stated that global progress towards remedying desertification is failing with the percentage of degrading land estimated to have increased from 15% in 1991 to 24% in 2008. To date, only two indicators are agreed for universal reporting^59^,^60^ namely: (i) the number of people affected; and (ii) the amount of green vegetation cover. The problem is that the world’s vulnerable drylands lack green vegetation. The breakthrough provided by ASTER over previous satellite systems is its ability to: (i) map where clays are exposed at the surface (Fig. 1b); (ii) measure the particle size of these exposed areas (Fig. 2a); and (iii) identify the clay (and other^30^) mineral composition (Figs 1a and 2b). This mineralogically comprehensive EO capability will not just enable more accurate characterisation and thus understanding of the Earth’s regolith and its processes but also improve our ability to assess, monitor and manage clay loss by wind erosion^60^,^61^, reducing the risk of desertification and other dust related issues^11^,^12^,^13^,^16^,^17^,^18^.

The current ASTER global archive comprises over 2.8 million scenes which could be used to generate approximately six temporal mosaics (spanning the years 2000 to 2008) of the CC, CI, CWI and SI products for the entire Earth’s land surface (<80° latitude). Given that ASTER’s SWIR module ceased operating in 2008, then the CC, CI and CWI products are no longer a monitoring option though similar SWIR clay mapping is possible through access to Worldview-3 (WV-3) satellite data which was launched in August 2014. Importantly, WV-3 comprises the same SWIR-2 bands as ASTER (except for band 9 which impacts on the CWI product) but at higher spatial resolution (7.5 m pixel) and lower volumes/rates of spatial coverage. ASTER’s TIR module continues to operate such that monitoring using an SI type product remains a global opportunity. Similarly, the Japanese HIMAWARI-8 satellite launched in October 2014 is now in geostationary orbit over the equator at 140° East (includes full coverage of Australia and a large part of Asia) and is imaging multispectral data every 10 minutes, including bands similar to ASTER’s TIR bands 10 and 13 albeit at 2 km pixel resolution. Thus routine detection of the loss or gain of surface clay during large-area dust activity or flood events is possible. Unfortunately though, ASTER’s integrated VNIR + SWIR + TIR spectral band configuration has not been matched by either WV-3 or HIMAWARI-8 such that future global mapping and monitoring the mineral complexity of Earth’s regolith and related processes must wait for new satellite systems.

## Methods

### NGSA spectral measurements

From the 0–10 cm depth NGSA sample suite, 165 random samples were taken and measured for their reflectance signatures across the 0.4 to 20 μm wavelength. The TIR spectra were measured using an integrating sphere attached to a Bruker Vertex 70 instrument which provide directional-hemispherical reflectance. The SWIR spectra were measured using an Analytical Spectral Devices (ASD) Fieldspec Pro 3 spectrometer, which provides bi-directional reflectance only. These two types of spectral measurements were then convolved using the published ASTER band-pass response functions^62^ before their assessment for statistical correlations with the critical indicators^34^, namely: (i) LOI (~weathering intensity); and (ii) % sand and/or % clay (~erosion-deposition). In addition, these indicators were also tested against the CIA^33^ and geochemical analogues for gamma-ray spectrometry data^34^ (K, Th and U). Statistical assessments were based on establishing linear regressions, which required in some cases initially transforming either the independent or dependent variables to improve their linearity. In such cases, a suitable power transformation was applied to help establish a normal distribution to the data.

### ASTER Clay-Water Index

The ASTER “Clay-Water Index” (CWI) is designed to be sensitive to water, including water vapour and bound or unbound molecular water associated with surface clays and other minerals. The CWI measures the slope of SWIR reflectance wing to the fundamental stretching vibration (symmetric and asymmetric) absorption of water at ~2.9 μm. The CWI is calculated using:


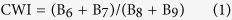


where B is the reflectance of a given ASTER band. The efficacy of the CWI is shown by its significant, linear correlations with the depth (R^2^ = 0.90) and area (R^2^ = 0.91) of the water combination (stretching plus bending vibration) absorption at ~1.9 μm for the full spectral resolution (~10 nm) ASD reflectance spectra of the NGSA 2 mm sample fractions^34^. Water associated with green vegetation and standing water could also contribute to the ASTER CWI signature, though the image processing in part limits this effect by masking in only those pixels that comprise detectable Al-clay absorption at 2.2 μm, namely:


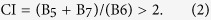


In theory, the CWI is also affected by water vapour as ASTER band 8 and especially band 9 can be significantly attenuated (27.6% difference between tropical and mid-latitude winter models) by water vapour whereas ASTER bands 6 and 7 span wavelengths of near complete atmospheric transmission (<4% difference between tropical and mid-latitude winter models). Water vapour is currently not corrected for in the Australian ASTER products, which helps explain why there are generally high CWI responses across the kaolinite-rich, tropical belt at lower latitudes (e.g. D in Fig. 2b). More arid areas appear to be less affected by variable water vapour. Ideally, water vapour should be corrected during ASTER data pre-processing before calculation of the CWI.

### Effects of vegetation cover

Mixing of both green and dry vegetation with exposed mineralogy in a given pixel results in lesser measurable mineral content than is actually present. One method developed for software defoliation^63^ first estimates the amounts of both green and dry vegetation which is then used to scale up the target mineral content. Unfortunately, even though ASTER has two bands specific to green vegetation, namely band 2 (chlorophyll absorption) and band 3 (near infrared reflectance plateau for green vegetation), it was not designed to also measure dry vegetation content, using for example cellulose absorption ~2.08 μm. ASTER does have spectral bands that span another dry vegetation component, namely lignin which has absorption at ~2.3 μm, though this features overlaps with many common minerals (e.g. carbonate, chlorite and amphibole) and thus of limited value. Thus unmixing ASTER mineral information of the contribution of green vegetation is theoretically possible though removing the effects of dry vegetation is more challenging. It is important to note though that the relatively limited spectral band configuration (compared with ASTER) of the Landsat series is used to isolate and the dry vegetation, green vegetation and bare soil components^64^.

The Version 1 ASTER geoscience products^30^ did not correct for the vegetation component, which explains why there is divergence of the NGSA field sample data with the ASTER CI and SI products (Figs 1b and 2a) outside of the arid zone. Importantly, mineral composition products, such as the ASTER CC appear to be independent of vegetative groundcover as indicated by its close spatial association with the NGSA surface sample CC results (Fig. 1a). The current Version 1 products include a green vegetation mask based on the ASTER index B_3_/B_2_ with the threshold set depending on the mineral product^30^. The ASTER Version 2 geoscience products currently being developed by CSIRO includes unmixing of the vegetation component/s.

## Additional Information

**How to cite this article**: Cudahy, T. *et al*. Satellite-derived mineral mapping and monitoring of weathering, deposition and erosion. *Sci. Rep*. **6**, 23702; doi: 10.1038/srep23702 (2016).

## Figures and Tables

**Table 1:** Least squares regression results for selected NGSA sample data (n = 165) where significant correlation is considered at the (i) 90% confidence interval to be where R^2^ > 0.33; (ii) 95% confidence interval to be where R^2^ > 0.39; and (iii) 99% confidence interval to be where R^2^ > 0.46.

	LOI	% sand	% clay
ASTER Al-clay composition *(CC)*	0.34	0.35	0.32
ASTER Al-clay content *(CI)*	0.04	0.04	0.10
ASTER water-clay *(CWI)*	0.21	0.40	0.45
ASTER Silica Index* (SI)	0.59	0.74	0.57
Al	0.32	0.61	0.40
Si	0.74	0.53	0.39
*CIA*	0.05	0.01	0.03
K	0.03	0.05	0.01
Th	0.05	0.12	0.05
U	0.12	0.11	0.02

Inverse relationships are underlined. ^*^Transformed to the power 0.32 to generate a normal distribution of data. Sand size is >20 μm but less than 2000 μm and clay size is <2 μm.

**Table 2:** Clay composition results measured from XRD analysis of suspended river sediments^35^ and their respective river catchments measured using the ASTER CC.

River system		River sample point latitude	River sample point longitude	Chlorite (%)	Montmorillonite (%)	Illite (%)	Kaolin (%)	Montmorillonite + illite (%)	Number of ASTER pixels in catchment	Mean of ASTER CC for catchment
Adelaide River	a	12°39.62′S	131°20.16′E	11	19	19	51	38	1047600	1.073
Ashburton River	b	22°32.63′S	115°29.92′E	7	6	50	37	56	58414500	1.07
Burdekin River	c	19°37.79′S	147°24.48′E	7	17	23	53	40	22234500	1.07
Cooper Creek	d	27°44.83′S	140°44.17′E	10	25	22	43	47	141275700	1.077
De Grey River	e	20°18.71′S	119°15.35′E	20	12	30	38	42	22589100	1.093
East Alligator River	f	12°25.51′S	132°57.92′E	10	24	15	51	39	2022300	1.03
Fitzroy River WA	g	17°43.65′S	123°38.45′E	11	18	36	35	54	31125600	1.074
Fitzroy River QLD	h	23°22.82′S	150°31.23′E	7	37	18	38	55	39049200	1.075
Fortescue River	i	21°17.60′S	116°08.62′E	22	9	25	44	34	19401300	1.0625
Gascoyne River	j	24°49.74′S	113°46.20′E	7	5	41	47	46	76700700	1.069
Jardine River	k	11°06.29′S	142°17.00′E	9	0	2	89	2	56700	0.97
Katherine River	l	14°27.72′S	132°15.52′E	9	17	22	52	39	1881900	1.024
Mangrove Creek, WA	m	21°57.94′S	113°56.59′E	12	3	54	31	57	1573200	1.1
Murchison River	n	27°49.68′S	114°41.38′E	11	2	34	53	36	90604800	1.045
Murray River Mildura	o	34°10.94′S	142°10.34′E	5	15	52	28	67	44934300	1.105
Murray River Swan	p	34°34.04′S	139°35.67′E	3	24	41	32	65	512198100	1.075
Murrumbidgee River	q	34°38.79′S	143°33.94′E	3	24	38	35	62	34847100	1.11
Normanby River	r	14°54.68′S	144°12.76′E	8	6	36	50	42	743400	1.044
Ord River	s	15°41.38′S	128°41.29′E	10	29	34	27	63	17855100	1.067
South Alligator River	t	12°39.46′S	132°30.34′E	9	22	19	50	41	1371600	1.025
Victoria River	u	15°36.61′S	130°24.07′E	5	42	21	32	63	17109000	1.072
*Least squares regressions (R*^*2*^*) for river sample XRD vs ASTER CC catchment clay composition*				<0.01	0.05	0.46	0.73	0.64		
